# Current status of lymph node micrometastasis in gastric cancer

**DOI:** 10.18632/oncotarget.17495

**Published:** 2017-04-27

**Authors:** Yang Zhou, Guo-Jing Zhang, Ji Wang, Kai-Yuan Zheng, Weihua Fu

**Affiliations:** ^1^ Tianjin Medical University General Hospital, Tianjin, China

**Keywords:** gastric cancer, lymph nodes metastasis, micrometastasis, molecular technique, minimally invasive surgery

## Abstract

Lymph node metastasis is one of the most important prognostic factors in patients with gastric cancer. An inadequate number of dissected lymph nodes is an independent risk factor affecting recurrence, even in patients who are node negative. Oddly, certain early-stage patients still experience recurrence or metastasis within a short time, even if they have undergone standard radical mastectomy. Many researchers have attributed these adverse events to lymph node micrometastasis (LNM), which is defined as a microscopic deposit of malignant cells of less than 2 mm in diameter. With the development of diagnostic tools such as immunohistochemistry and reverse transcription-polymerase chain reaction, the rate of detection of LNM has been constantly increasing. Although there is no clear consensus about risk factors for or the definitive clinical significance of LNM, the clinical impact of LNM is remarkable in gastric cancer. For minimally invasive treatment in particular, such as endoscopic submucosal dissection and laparoscopic surgery, accurate diagnosis of LNM is regarded as the potential key to maintaining the balance between curability and safety. This review provides an overview of the definition, detection and significance of LNM in gastric cancer. We also summarize several attention-drawing controversies regarding the treatment of patients who may have LNM.

## INTRODUCTION

Gastric cancer remains one of the most frequently occurring malignancies. It is the third leading cause of cancer-related deaths worldwide, and its prevalence is increasing [1]. Generally, patients with node-negative gastric carcinoma have a good prognosis compared with patients who are node positive [2]. In China, the many new patients who are diagnosed at advanced stages have missed the best opportunity for curative surgery [3]. Even for patients without node metastasis, there is a certain recurrence rate after extensive lymphadenectomy [4]. Historically, representative sections from removed nodes are used in histological examination, and lymph node micrometastasis (LNM) that is not detected by routine pathologic examination may be identified in multiple sections of lymph nodes. The detection of LNM has been exposed as the weakness of histological examination, and this has prompted researchers to seek a more sensitive way to characterize micrometastasis-related tumour cells. Due to technological developments, such as immunohistochemistry (IHC) and reverse transcription-polymerase chain reaction (RT-PCR), the rate of diagnosis of micrometastasis has increased significantly. Comparatively speaking, IHC can offer satisfactory accuracy for the detection of LNM, whereas RT-PCR is more sensitive but may offer false-positive results caused by various sources of biological contamination. LNM has increasingly been the focus of research based on these diagnostic tools. However, there have been many controversies about the impact of LNM, such that no consensus on the clinical treatment or significance of micrometastatic node involvement in patients with gastric cancer has been reached. In recent years, with the rise of endoscopic submucosal dissection (ESD), laparoscopic surgery and other minimally invasive treatments have been widely performed in consideration of postsurgical quality of life (QOL). However, LNM may have an unfavourable influence on recurrence after these unconventional treatments, and the need to balance the relationship between QOL and safety is making research on micrometastasis more urgent.

## DEFINITION OF MICROMETASTASIS

LNM was initially defined as a microscopic deposit of malignant cells of less than 2 mm in diameter [5]. This deposit has the ability to escape immune supervision and to progress to macroscopic malignant growth. With deeper study, the Union for International Cancer Control (UICC) redefined single tumour cells or cell clusters measuring ≤ 0.2 mm in the greatest dimension as “isolated tumour cells” (ITCs) because of their different biological behaviour and size. In other words, micrometastasis was defined as referring to tumour cell clusters between 0.2 mm and 2 mm in the greatest dimension [6]. The effort was aimed at accurate staging and distinguishing the entities’ different biological behaviours. According to the 7th TNM classification by the UICC, first, LNM should be considered in node staging of gastric cancer [7]. Patients with LNM are staged as pN1(mi), and patients with ITCs in the lymph nodes are staged as pN0(i+). Moreover, if micrometastasis can be detected only by RT-PCR, its N stage should be determined as pN0(mol+) [8]. Second, ITCs do not show evidence of metastatic activity, such as proliferation or penetration of the vascular system, compared with LNM. LNM is not equal to lymph node metastasis mainly because of different outcomes. The lack of neovascularization means that tumour sizes cannot exceed 3 mm. At the same time, the cells may be in a state between proliferation and death. VEGF-C is considered the key factor in breaking dormancy [9]. The uncertain mechanisms of and conditions for LNM growth are responsible for the very different conclusions reported from different centres.

## DETECTION OF MICROMETASTASIS

### Serial sectioning

Serial sectioning has been applied in several methods for LNM detection, and subsequently, the rate of detection of nodal micrometastasis markedly increased compared with that for HE staining, which is limited because many small tumour cell clusters may be overlooked [10]. Approximately 40% of patients classified as pN0 according to traditional HE staining methods have nodal micrometastasis. With narrower slice intervals, the detection rate gradually increases. However, the weaknesses of a heavy workload and low efficiency have limited this approach's application, so it has been supplemented with or replaced by more sensitive procedures.

### Immunohistochemical staining

Small lesions not visible by routine pathologic examination can be detected by IHC, which has been utilized as a standard tool for detecting LNM in gastric cancer. Many studies have confirmed significantly higher sensitivity for immunohistochemical methods than for HE staining. Meanwhile, cytokeratin (CK) AE1/AE3 and CAM5.2 have been identified as reliable markers of epithelial cells in lymph nodes [11]. These markers’ biggest advantage is their ability to distinguish a single tumour cell from small clusters of tumour cells using morphological distinctions, and this technique has been shown to be invaluable in retrospective studies. In 2003, Matsumoto et al. introduced a rapid IHC technique to diagnose LNM within 30 minutes during operations for gastric cancer [12]. The incidence of LNM according to different reports is diverse. For T1-stage cases, Maehara et al. [13] and Kashimura et al. [14] reported that the incidence of LNM was 23.5% and 23.4%, respectively. However, Kim et al. [15] reported that the incidence was only 10% in pT1N0 patients. For T2-stage cases, according to a report by Kikuchi et al., the rate was 43.2% [16]. Considering these research results, the incidence of LNM seems to be related to deep tumour invasion. The lack of consensus on this topic is a problem, and more experimentation is needed to evaluate the issue. The number of lymph node sections required is another issue in the application of IHC. According to a study that included 98 patients with colorectal cancer, Noura et al. [17] argued that at least five sections should be assessed. There have been no related studies on gastric cancer. Compared with RT-PCR, the advantages of immunohistochemical methods are their availability, their intuitive results and their lower costs, but these methods are more likely to be affected by the experimental techniques and conditions used, so they tend to be less accurate [18].

### RT-PCR assay

The greatest advantage of RT-PCR is its sensitivity, such that 1 tumour cell in 1×10^7^ cells can be detected [19]. The introduction of RT-PCR increased the rate of detection of LNM to a level higher than that achieved using IHC [20]. The amplification of gene transcripts associated with occult tumour cells is a reason for the higher sensitivity of RT-PCR. The key in using the RT-PCR method is to select an appropriate target marker. Tissue-specific genes, such as CEA, tend to be used [20] because no gastrointestinal cancer-specific marker genes have been discovered and because CEA is expressed in most cancers and in normal gastrointestinal cells. Generally, the rate of detection of LNM is greater than 30%. Jagric et al. [21] published a study in which micrometastasis was detected in 40% of patients. History justifies our confidence in the method but also shows that it is not fool proof. Marker-related pseudogenes, incorrect transcription of genes and various sources of biological contamination are responsible for false positives. In contrast, heterogeneous expression of a target marker can lead to false negatives. Although RT-PCR sometimes offers false results, this technique is still considered to be the most accurate option for the detection of LNM. Kubota et al. [22] reported that the detection rates of RT-PCR and IHC were 9.9% and 3.6%, respectively. Similarly, the respective outcomes reported by Arigami et al. were 31.3% and 11.3%. To avoid false detection results, a multiplex RT-PCR assay has been recommended for application [23]. In the future, this molecular system is expected to become an intraoperative diagnostic tool for the rapid detection of LNM. For this reason, the system may be regarded as a protective measure for patients undergoing minimally invasive surgery with personalized lymphadenectomy (Table 1).

**Table 1:** IHC and RT-PCRl studies in gastric cancer patients with node negativity diagnosed by HE staining

Year	Study	No.of patients	Pathologic results by HE	Methods	Target markers	Incidence(%)
1996	Maehara et al.[13]	34	pT1N0	IHC	CK(CAM5.2)	23.5%
1999	Kashimura et al.[14]	47	pT1bN0	IHC	CK(CAM5.2)	23.4%
2000	Harrison et al.[44]	25	pT1-4N0	IHC	CK(CAM5.2)	36.0%
2000	Cai et al.[45]	69	pT1bN0	IHC	CK(CAM5.2)	24.6%
2001	Fukagawa et al.[34]	107	pT2-3N0	IHC	CK(AE1/AE3)	35.5%
2001	Morgagni et al.[46]	139	pT1N0	IHC	CK(MNF 116)	17.3%
2001	Nakajo et al.[27]	67	pT1-3N0	IHC	CK(AE1/AE3)	14.9%
2002	Lee at al.[47]	41	pT1N0	IHC	CK(AE1/AE3)	24.4%
2002	Yasuda et al.[30]	64	pT2-4aN0	IHC	CK(CAM5.2)	31.3%
2002	Choi et al.[48]	88	pT1bN0	IHC	CK(35βH11)	31.8%
2003	Morgagni et al.[28]	300	pT1N0	IHC	CK(MNF 116)	10.0%
2006	Miyake et al.[49]	120	pT1N0	IHC	CK(AE1/AE3)	22.5%
2007	Yonemura et al.[50]	308	pT1-4N0	IHC	CK(AE1/AE3)	12%
2008	Kim et al.[51]	184	pT1-4aN0	IHC	CK(AE1/AE3)	16.8%
2008	Ishii et al.[52]	35	pT1b-2N0	IHC	CK(O.N>352)	11%
2011	Cao et al.[32]	160	pT1N0	IHC	CK(AE1/AE3)	21.3%
2011	Wang et al.[53]	191	pT1-3N0	IHC	CK(AE1/AE3)	28.3%
2012	Ru et al.[29]	45	pT1-4N0	IHC	CK(19),CD44v6	33.3%
2001	Okada et al.[54]	24	pT1-4aN0	RT-PCR	EA,CK20,	41.7%
2002	Matsumoto et al.[20]	50	pT1-4N0	RT-PCR	CEA	28%
2005	Arigami et al.[55]	80	pT1-3N0	RT-PCR	CEA	31.3%
2006	Sonoda et al.[56]	33	pT1N0	RT-PCR	MUC 2,TFF 1	33.3%
2007	Wu et al.[57]	10	-	RT-PCR	CK20	20%

## CLINICAL SIGNIFICANCE OF NODAL MICROMETASTASIS

### LNM and the staging system

The staging of gastric cancer is an important and controversial issue. The significance of micrometastasis is often obscured by macrometastasis. Many researchers have focused on the detection of LNM in patients with pN0-stage cancer and regard it as an early event indicating the possibility of clinical metastasis. In the past, researchers attached importance to macrometastasis and disregarded micrometastasis. However, chronic negligence of LNM may have led to underestimation of the disease state. According to the 7th edition of the American Joint Committee on Cancer system [24], the N stage is determined by the number of metastatic nodes [24]. Furthermore, when the number of metastatic nodes exceeds 15, the detection of LNM is unnecessary because the situation has already indicated the most advanced N stage. However, the detection of LNM could improve staging when the number of metastatic nodes is less than 15. In other words, the N stage may vary with the detection of LNM [25]. Lee et al. [26] reported that the rate of detection of LNM was 32.4% in 482 patients and that the patients with LNM had a worse 5-year survival rate. The authors surmised that ignorance of LNM, which may have the same prognostic value as lymph node macrometastasis, causes the tumour stage to be underestimated, resulting in an overly optimistic conclusion regarding the disease state. Many institutes have not recognized the value of LNM in the staging process. Accounting for this problem, Lee et al. [26] recommended a new staging system that included LNM. Compared with conventional staging systems, the researchers found that in their staging system, the prognostic difference between stages N2 and N3a was more distinct. This exciting result implied that LNM could affect staging in patients with advanced gastric cancer as well as in patients with early gastric cancer.

### The prognostic value of LNM

Results for the predictive value of LNM have varied between different studies [27, 28]. Many researchers have suggested that patients with LNM have a poor prognosis [29, 30]. The prognosis of gastric cancer is relevant to the tumour stage, and the discovery of LNM often means that the stage of the tumour improved spontaneously but that the prognosis is poor [31]. Using gastric cancer-related death as the end point, Li et al. [31] found that the 5-year survival rates of patients with LNM were significantly poorer than those of patients without LNM (HR=2.81, 95% CI 1.96~4.02). Lee et al. [26] and Cao et al. [32] came to the same conclusion. In the first meta-analysis of the influence of LNM on the prognosis of gastric cancer, the researchers declared that in patients with gastric carcinoma, LNM was associated with a higher recurrence rate [33], but LNM should not be seen as the gold standard for prognosis evaluation in patients with gastric cancer. Conversely, certain researchers have suggested that LNM does not affect the prognosis of patients. A study from the National Cancer Center Hospital in Tokyo showed that the presence of nodal micrometastasis does not correlate with survival in patients with advanced gastric cancer. A study including 300 patients with stage T_1_N_0_ cancer also showed that LNM did not affect the survival rate of patients at 5 years or 10 years [28]. The host immune response was considered to be one of the reasons for this phenomenon. Another study comprising 107 patients with T_2_N_0_M_0_ cancer also showed that the 5-year survival rates of patients with or without LNM were 89% and 94%, respectively; the difference was not statistically significant [34]. The prognostic value of LNM remains controversial, so an increasing number of researchers have aimed to explain the reasons for this variation by exploring the conditions required for micrometastasis progression to macroscopic malignant growth. Our knowledge of the natural growth history is increasing but remains limited. We believe that the heterogeneity of LNM among patients is responsible for the controversy. The sources of heterogeneity may include different approaches to staging, different therapeutic regimens, and different physical states, among others. The effects of these confounding factors on LNM make it difficult to identify the most important factor. In particular, if the effects of these differences could be eliminated, studies might be able to reach a relatively reliable conclusion.

## CLINICAL TREATMENT STRATEGIES

### Traditional surgery

Japanese researchers have emphasized the significance of D2 lymphadenectomy for patients with gastric carcinoma, whereas Western researchers have suggested that it does not show a survival advantage compared with D1 radical surgery [35]. In Asia in particular, a large number of studies have confirmed the superiority of a D2 operation, which is considered the standard surgical procedure for gastric cancer [36]. The phenomena of micrometastasis and jumping metastases also seem to suggest that reduction of the scope of the operation should be performed with caution. As previously described, the incidence of LNM in gastric cancer is higher than 10%, even in both mucosal and submucosal cancers. As a potential clinical metastasis source, LNM can become a source of recurrence once micrometastasis is activated. The existence of LNM also increases the rationality of the expansion of lymph node dissection, or at least the scope of the operation should not be easily reduced, especially in the diffuse type of gastric cancer. A number of researchers have declared that extensive lymphadenectomy can improve the survival rate of gastric cancer patients without lymph node metastasis, which may be related to the reduction of the presence of LNM. Meanwhile, many researchers have argued that LNM is not equivalent to clinical metastasis and may be cleared depending on the defence system and immune system capabilities. Even if the first line of natural defence is broken, LNM may still be effectively inactivated with the help of preoperative or postoperative adjuvant chemotherapy. Certain radical researchers have even suggested that extensive lymphadenectomy is only beneficial to improve the accuracy of staging [37].

### Endoscopic resection

In recent years, ESD has been increasingly suggested to replace radical resection in the treatment of early gastric cancer because of its better postoperative QOL [38]. The implementation of ESD must meet two conditions: (1)there is no lymph node metastasis, and (2)the disease focus can be excised all at once. The evaluation of lymph node status is the most important precondition [39]. An accurate intraoperative evaluation is necessary when performing minimally invasive surgery because the clinical impact of metastasis is contentious, i.e., ESD can be applied when lymph node metastasis is not expected [40]. Chinese researchers have found that the tumour invasion depth, lymph node metastasis and vascular invasion may affect the prognosis of patients with early gastric cancer but that only lymph node metastasis is an independent prognostic factor. If the treatment can be performed based on accurate assessment of lymph node status, the relationship between postoperative QOL and the safety of treatment can be well balanced. However, Japan's Guide for the Treatment of Gastric Cancer (April 2010 Edition) [41] continues to retain the expanded ESD/endoscopic mucosal resection (EMR) indications. At the same time, the guide emphasizes that more clinical trials are needed due to the lack of sufficient clinical evidence. If we consider the concept of LNM, these terms cause more concerns regarding the techniques’ application. For all diagnostic tools, none had an accuracy greater than 90%, and pathologic examination remains the gold standard for assessing lymph node metastases. Thus, EMR/ESD bears the risk of missing lymph node metastases. The results of research on the accuracy of ESD from Japan and South Korea were significantly more optimistic than those from Europe and the United States; this may be related to differences in the ESD pointer, the technique and other aspects. Japan's National Cancer Center conducted a massive study in which 1,955 people with early gastric cancer were included, and it was found that the 5-year survival rates for ESD and radical mastectomy were not significantly different [42]. Another large sample study including 1,485 patients with early-stage cancer showed that the 5-year survival rate for ESD was 92.4%. There was no statistically significant difference between ESD and radical mastectomy operations. In addition, certain Western studies have shown that the safety of ESD is relatively poor. In contrast, the recurrence rate is significantly lower in patients with early gastric cancer without lymph node metastasis who underwent standard radical mastectomy. With progress in detection, the rate of detection of LNM after operation has greatly improved. In addition, early gastric cancer has its own special metastatic form and mechanism [43]. Therefore, considering the concept of LNM and endoscopic treatment, performing local resection of tumours instead of expanding lymph node dissections is controversial. The risk of ESD/EMR should also be re-evaluated. To answer these questions, a large number of studies on the mechanisms of and conditions for LNM growth are also required. Because of issues with operating techniques and security, among other issues, ESD technology has not yet been promoted in Europe or the United States.

## CONCLUSIONS

Nearly all the recent studies on lymph node metastasis have been retrospective analyses. There is a lack of studies on the mechanisms of LNM proliferation. This is precisely the key to breaking through the bottleneck in current research. With in-depth study of its biological behaviour, we believe that there will be a more reasonable and individualized comprehensive treatment plan for patients with gastric cancer, and especially patients with early gastric cancer. Looking forward, minimally invasive surgery may be safely performed and may achieve satisfactory results with regard to the balance between QOL and curability.
